# Single-particle imaging without symmetry constraints at an X-ray free-electron laser

**DOI:** 10.1107/S205225251801120X

**Published:** 2018-09-18

**Authors:** Max Rose, Sergey Bobkov, Kartik Ayyer, Ruslan P. Kurta, Dmitry Dzhigaev, Young Yong Kim, Andrew J. Morgan, Chun Hong Yoon, Daniel Westphal, Johan Bielecki, Jonas A. Sellberg, Garth Williams, Filipe R.N.C. Maia, Olexander M. Yefanov, Vyacheslav Ilyin, Adrian P. Mancuso, Henry N. Chapman, Brenda G. Hogue, Andrew Aquila, Anton Barty, Ivan A. Vartanyants

**Affiliations:** a Deutsches Elektronen-Synchrotron DESY, Notkestrasse 85, Hamburg D-22607, Germany; b National Research Centre ’Kurchatov Institute’, Akademika Kurchatova pl. 1, Moscow 123182, Russia; c Center for Free Electron Laser Science (CFEL), Notkestrasse 85, Hamburg 22607, Germany; d European XFEL GmbH, Holzkoppel 4, Schenefeld 22869, Germany; e Linac Coherent Light Source, SLAC National Accelerator Laboratory, 2575 Sand Hill Road, Menlo Park, CA 94025, USA; f Laboratory of Molecular Biophysics, Department of Cell and Molecular Biology, Uppsala University, Sweden; g Biomedical and X-Ray Physics, Department of Applied Physics, AlbaNova University Center, KTH Royal Institute of Technology, Stockholm SE-106 91, Sweden; h Brookhaven National Laboratory, 98 Rochester St, Shirley, NY 11967, USA; i NERSC, Lawrence Berkeley National Laboratory, Berkeley, CA, USA; j Biodesign Center for Immunotherapy, Vaccines, and Virotherapy, Biodesign Institute at Arizona State University, Tempe 85287, USA; k Biodesign Center for Applied Structural Discovery, Biodesign Institute at Arizona State University, Tempe, AZ 85287, USA; l Arizona State University, School of Life Sciences (SOLS), Tempe, AZ 85287, USA; m National Research Nuclear University MEPhI (Moscow Engineering Physics Institute), Kashirskoe shosse 31, Moscow 115409, Russia

**Keywords:** single-particle imaging, three-dimensional virus reconstructions, XFELs

## Abstract

The analysis of a single-particle imaging (SPI) experiment performed at the AMO beamline at LCLS as part of the SPI initiative is presented here. A workflow for the three-dimensional virus reconstruction of the PR772 bacteriophage from measured single-particle data is developed. It consists of several well defined steps including single-hit diffraction data classification, refined filtering of the classified data, reconstruction of three-dimensional scattered intensity from the experimental diffraction patterns by orientation determination and a final three-dimensional reconstruction of the virus electron density without symmetry constraints. The analysis developed here revealed and quantified nanoscale features of the PR772 virus measured in this experiment, with the obtained resolution better than 10 nm, with a clear indication that the structure was compressed in one direction and, as such, deviates from ideal icosahedral symmetry.

## Abstract 2

Data-processing workflow for single-particle imaging experiments at X-ray free-electron lasers is presented. The analysis developed here revealed nanoscale features of the PR772 virus with a resolution better than 10 nm and without any symmetry constraints.

## 1. Introduction   

Single-particle imaging (SPI) performed using hard X-ray free-electron lasers (XFELs) (Altarelli *et al.*, 2006 ▸; Emma *et al.*, 2010 ▸; Ishikawa *et al.*, 2012 ▸) was proposed more than a decade ago (Neutze *et al.*, 2000 ▸; Miao *et al.*, 2001 ▸; Gaffney & Chapman, 2007 ▸) as a method of determining the structure of individual biological samples from viruses to single molecules in their native environment. This is different from cryo-electron microscopy (Bai *et al.*, 2015 ▸), where single biological particles have to be preserved at liquid nitrogen temperatures to determine their structure. Importantly, XFELs may provide another dimension for the study of biological systems, namely, time evolution in pump-probe experiments on extremely small time scales.

XFELs generate pulses with ultra-high brilliance and high spatial coherence on the femtosecond time scale that is a prerequisite for the success of SPI at XFEL sources. The first experiments performed on protein nanocrystals (Chapman *et al.*, 2011 ▸) and single viruses (Seibert *et al.*, 2011 ▸) at the Linac Coherent Light Source (LCLS) were very promising and raised high expectations in the community. Significant progress in the field and several successful structure recoveries of biological samples were reported later (Kimura *et al.*, 2014 ▸; Hantke *et al.*, 2014 ▸; van der Schot *et al.*, 2015 ▸; Ekeberg *et al.*, 2015 ▸).

At the same time it was realized that the target of high-resolution (potentially atomic resolution) structure determination of biological particles at XFELs is more challenging to achieve than initially anticipated. This lead to formation of the SPI initiative at LCLS and an international team with the goal to further progress single-particle imaging with XFELs (Aquila *et al.*, 2015 ▸).

**Figure 1:**
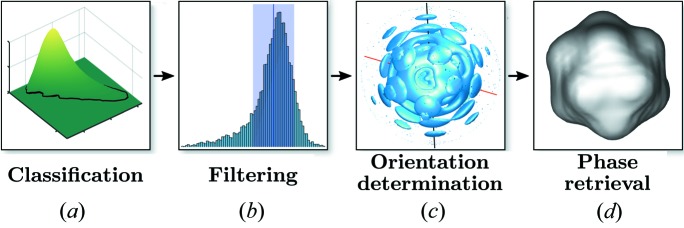
Workflow of the SPI experiment towards single-particle reconstruction: (*a*) single-hit classification of the initial data, (*b*) refined filtering of the classified data, (*c*) orientation determination, (*d*) particle reconstruction using phase retrieval.

Here we present the results of the structure determination of the PR772 virus from experimental data collected using soft X-ray pulses at LCLS as part of the SPI initiative (Reddy *et al.*, 2017 ▸). Complementary to recent work published on the same experimental data (Hosseinizadeh *et al.*, 2017 ▸; Kurta *et al.*, 2017 ▸), we chose a different approach for the analysis using the workflow illustrated in Fig. 1 ▸. We implemented a strategy consisting of several steps previously outlined (Gaffney & Chapman, 2007 ▸). Preliminary filtering (Reddy *et al.*, 2017 ▸) resulted in an initial data set that was further refined by an advanced classification approach (Bobkov *et al.*, 2015 ▸) and additional filtering procedures. Next, an orientation determination procedure  (Loh & Elser, 2009 ▸) was applied to determine the full three-dimensional intensity distribution originating from the virus particle. The final step of reconstruction from this intensity distribution was performed to obtain the three-dimensional structure of the PR772 virus. Importantly, symmetry constraints were not used during the reconstruction. A detailed description of all steps leading to a final reconstruction of the particle structure is presented in this work.

## 2. Experiment and initial data processing   

The experiment was conducted using the Atomic Molecular Optics (AMO) instrument at the LCLS. The PR772 bacterio-phage with a diameter of about 70 nm was chosen. The sample was aerosolized by a Gas Dynamic Virtual Nozzle (GDVN), and the particle stream was focused by an aerodynamic lens stack into the XFEL beam which had a photon energy of 1.6 keV (wavelength 0.775 nm). The far-field diffraction patterns from randomly oriented particles were measured by a pnCCD detector (Strüder *et al.*, 2010 ▸). The details of the raw data processing, sample preparation and experimental conditions were recently reported (Reddy *et al.*, 2017 ▸).

The data analysis consisted of multiple steps of classification and filtering; useful single-hit diffraction patterns were defined as those where only a single virus was present in the XFEL beam. This single-hit class contains the most valuable data for structure determination by SPI.

**Figure 2:**
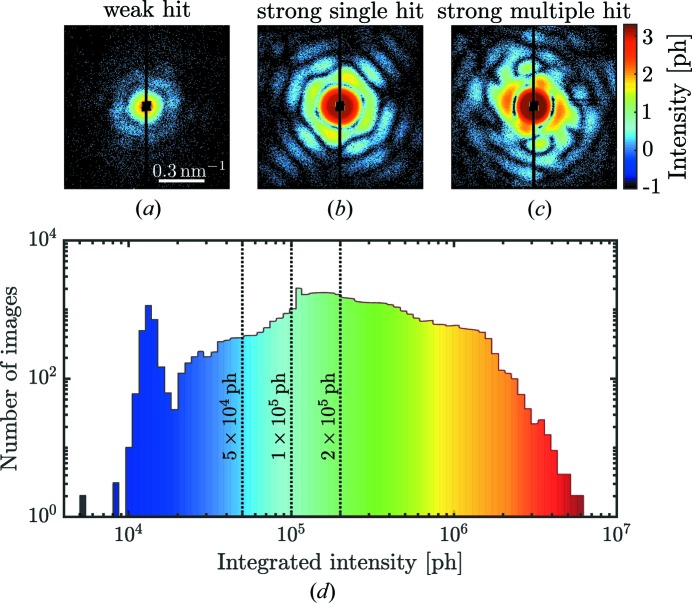
(*a*)–(*c*) Typical diffraction patterns collected during the SPI experiment with (*a*) typical candidates for weak single hits, (*b*) strong single hits and (*c*) multiple hits (all diffraction patterns are shown on a logarithmic scale). (*d*) Histogram of diffraction images as a function of the integrated intensity. Three dashed threshold lines mark the transient region between weak and strong hits passing our intensity filter. Diffraction patterns in (*a*) and (*c*) belong to blue and red regions in (*d*), respectively. The diffraction pattern in (*b*) belongs to the central region in (*d*).

The full data set that includes all XFEL pulses consists of the initial number of Set_3M_ = 2 976 568 images (Reddy *et al.*, 2017 ▸). From this data set, Set_44k_ = 44 039 was selected. This hit-selection procedure was based on a χ-squared metric previously described by Reddy *et al.* (2017 ▸), which selects images with strong scattering (for example, single or multiple particles). The detector images were then downsampled by 4 × 4 and the detector intensities were converted to photon counts. Typical diffraction patterns from Set_44k_ are shown in Figs. 2 ▸(*a*)–2(*c*). Single-hit diffraction patterns providing Set_14k_ = 14 772 images were further selected using the diffusion-map approach described by Reddy *et al.* (2017 ▸).

Contrary to the above approach, we first sorted the diffraction patterns from Set_44k_ by their integrated intensities [see the histogram in Fig. 2 ▸(*d*)]. Weak hits with an integrated intensity less than 5 × 10^4^ ph (photons) were excluded from further single-hit analysis since their classification was difficult to determine with sufficient accuracy. We also identified very strong hits with integrated intensity higher than 2 × 10^6^ ph. These consisted predominantly of multiple hits that were mostly filtered out in the next step. Since the transition between strong and weak hits was smooth, we used different thresholds for the total number of photons counted in a diffraction pattern as an initial filtering step [see vertical lines in Fig. 2 ▸(*d*)]. The respective number of diffraction patterns for each set is shown in Table 1 ▸. This selection was then used in the single-hit classification by the principal component analysis (PCA) technique explained below.

## 3. Classification by the principal component analysis   

The novel and most important filtering step used in this work is based on the classification by the PCA technique (Bobkov *et al.*, 2015 ▸). From our experience, direct application of PCA methods to experimental diffraction patterns is not efficient and single-hit classification is hindered substantially as a result of different particle orientations and incident fluctuating X-ray-pulse intensities. Therefore, we compress each diffraction pattern into a feature vector (FV), which describes the diffraction patterns in relation to the real-space structure of a particle (see Supporting Information for the definition of the FV). The FV compression (resulting FV consists of 50 components) is based on X-ray cross-correlation analysis (XCCA) (Wochner *et al.*, 2009 ▸; Altarelli *et al.*, 2010 ▸; Kurta *et al.*, 2016 ▸).

**Figure 3:**
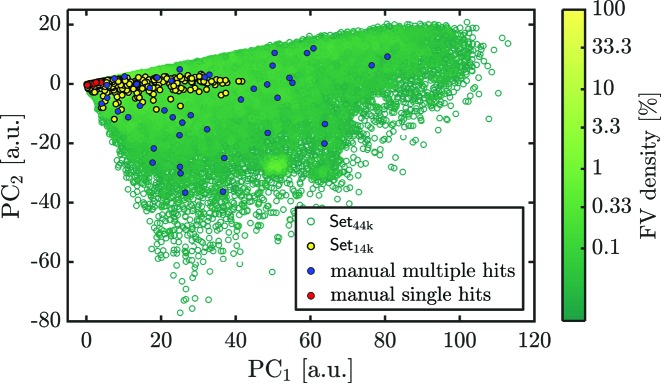
Projection of feature vectors onto the PCA plane. Each dot corresponds to a diffraction pattern. The green empty dots represent diffractions patterns of Set_44k_ and the yellow dots represent single hits of Set_14k_. The manually classified patterns are marked by red (single hits) and blue dots (multiple hits).

The PCA method is then applied to the set of FVs instead of the full diffraction patterns to separate single hits from other classes. With the PCA we projected the FV values (representing the diffraction patterns) onto a plane of the first and second principal components (PC_1_ and PC_2_) as shown in Fig. 3 ▸. Diffraction patterns with similar features cluster in a distinct region on the plane. Diffraction patterns with large differences are spread widely throughout the plane. We manually selected about 50 single-hit diffraction patterns similar to the one shown in Fig. 2 ▸(*b*). These are marked as red dots on the PCA plane in Fig. 3 ▸. We also selected about 50 diffraction patterns similar to the multiple-hit diffraction shown in Fig. 2 ▸(*c*) and shown as blue dots on the PCA plane in Fig. 3 ▸.

In the PCA plane we discovered a densely packed region which we attributed to single hits. For comparison, we also show the initial data Set_44k_ as green empty dots and another single-hit selection of Set_14k_ as yellow dots from the paper by Reddy *et al.* (2017 ▸). From these observations, we suggest that our PCA technique with prior FV compression may be useful in selecting single hits from SPI experimental data.

**Figure 4:**
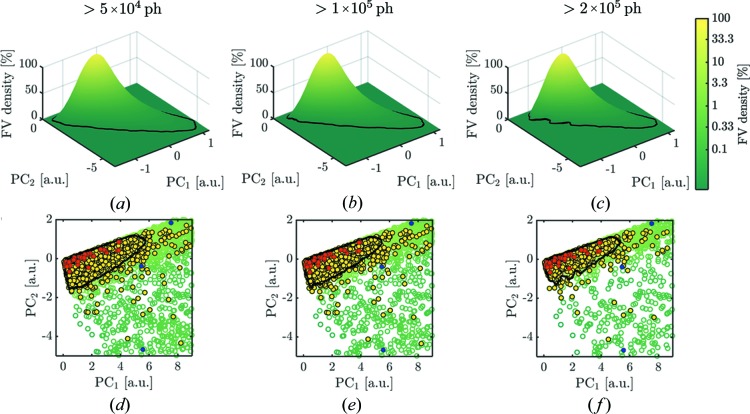
(*a*)–(*c*) FV densities on PCA planes for different intensity thresholds (number of hits in Table 1 ▸). (*d*)–(*f*) Projection view of the PCA densities with manually classified single-hit patterns shown as red dots. Blue and yellow dots correspond to the same selections as in Fig. 3 ▸. The black contour level corresponds to 3.3% of the maximum value of the PCA density (selected hits in Table 1 ▸). For low-intensity thresholds, the black contour contains a region that is not clearly represented by the manual single hit selection (*a*), (*d*) and (*b*), (*e*). The manual hit selection is most precisely matched by data Set_10k_^PCA^ within the contour line for an intensity threshold at 2 × 10^5^ ph in (*c*) and (*f*).

The PCA densities are visualized as three-dimensional plots to show the dense single-hit areas [Figs. 4 ▸(*a*)–4(*c*)] and enlarged PCA plane plots [Figs. 4 ▸(*d*)–4(*f*)] with red and blue dots for the single- and multiple-hit indicators, respectively. Each column of Fig. 4 ▸ represents the data selected by the integrated photon count at different thresholds. Separation of single hits from other data had to be done carefully because of the rather smooth transition between principal components of single hits and multiple hits. Here we used the area at 3.3% of the maximum value of the FV density which is visualized as a black contour line in the PCA density plots in Fig. 4 ▸. The result of all selections is summarized in Table 1 ▸. For the threshold of 2 × 10^5^ ph, the best single-hit selection was expected because the black contour line most tightly encircles the manually selected single hits. A further increase of the integrated intensity threshold was not desired as this could substantially decrease selected amount of data. Within the black contour line Set_10k_^PCA^ = 10 082 diffraction patterns were assigned as single-hit candidates.

## 4. Filtering of particle-size distribution   

**Figure 5:**
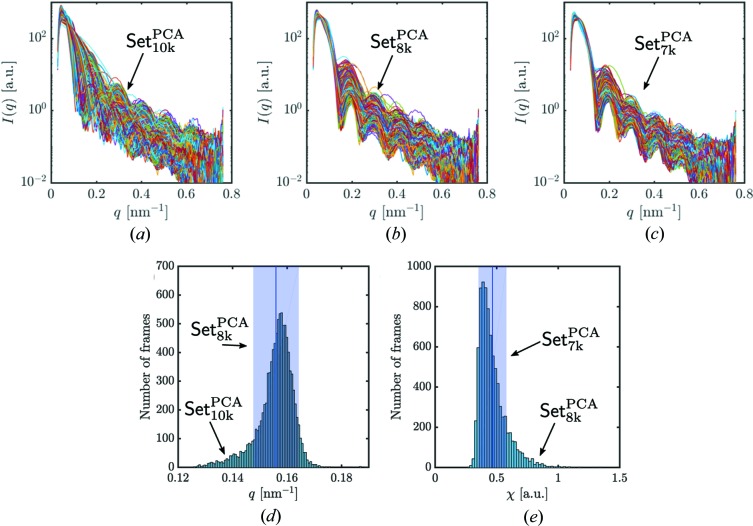
Power spectral density (PSD) for distinct data sets obtained at different stages of particle-size filtering. (*a*) PSDs for the data Set_10k_^PCA^ of diffraction patterns. (*b*) PSDs after the size-distribution filtering for Set_8k_^PCA^. (*c*) PSDs after using a restricted fit quality range for Set_7k_^PCA^. (*d*) Histogram of positions of the first minimum from data Set_10k_^PCA^ used for the size filtering. Diffraction patterns inside the blue box (±1 r.m.s. around the mean value) were selected for further analysis. (*e*) Histogram of the PSD fit quality χ. Diffraction patterns inside the blue box (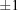 r.m.s. around the mean value) were selected for further analysis.

The particle size can be estimated, for example, from the power spectral density (PSD), 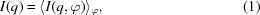
which is the intensity averaged over the azimuthal angle φ. Here *q* is the magnitude of the momentum transfer vector. We show the PSD of all individual diffraction patterns contained in Set_10k_^PCA^ obtained by the PCA technique in Fig. 5 ▸(*a*). From visual inspection we find clear outliers that are not filtered out by the PCA technique. In order to apply further filtering stages we approximated the diffraction patterns by the form factor of a sphere (Pedersen, 1997 ▸),
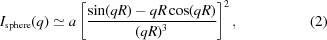
where *a* is a constant and *R* is the sphere radius.

The *q*-value distribution of the first characteristic minimum was determined by fitting each PSD with equation (2). The histogram of positions of the first minimum from Set_10k_^PCA^ has a broad range of *q* values [Fig. 5 ▸(*d*)]. This suggests that Set_10k_^PCA^ still contains some diffraction patterns which correspond to particles of different sizes. In order to narrow the size distribution, we selected ±1 r.m.s. value around the mean value of the distribution in Fig. 5 ▸(*d*) and obtained Set_8k_^PCA^ containing 8459 diffraction patterns (see Table 1 ▸). However, the PSDs of Set_8k_^PCA^ still show some outliers [Fig. 5 ▸(*b*)]. Besides the minimum position, we also exploited the quality of each fit to the PSDs. The fit quality was defined as
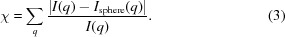
By its definition it compares the fitted data with the measured data. We used ±1 r.m.s. value around the mean value of the fit quality histogram [see Fig. 5 ▸(*e*)] as the last filtering step and obtained the final single-hit selection of Set_7k_^PCA^ containing 7 303 diffraction patterns (see Table 1 ▸). After this final filtering step, the PSD for our final selection is cleaned from the obvious outliers as seen in Fig. 5 ▸(*c*).

**Figure 6:**
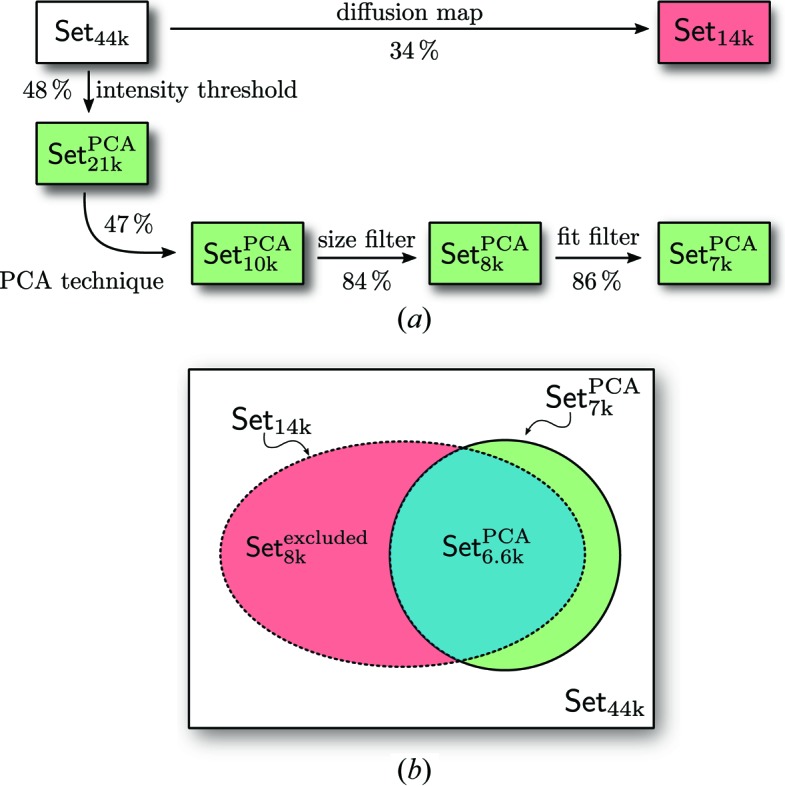
(*a*) Data workflow and filtering stages. The red box indicates the data selection provided by Reddy *et al.* (2017 ▸) and the green boxes show the selection from the PCA technique. (*b*) Schematic of data set relations with the intersection shown in blue.

Multiple filtering steps have been taken to obtain a well defined data set that comprises predominantly single hits with a narrow size distribution of particles. A block diagram giving an overview of the selection steps is shown in Fig. 6 ▸(*a*). The PCA technique as the main filtering stage passes 47% after the intensity threshold. The contributions from the other filtering steps are given in Fig. 6 ▸(*a*).

We compared the data selection described here with the initial single-hit selection (Reddy *et al.*, 2017 ▸). Fig. 6 ▸(*b*) clearly shows that a large part of Set_6.6k_^PCA^, with 6 677 diffraction patterns, is shared between our data (Set_7k_^PCA^) and the data Set_14k_ previously selected by Reddy *et al.* (2017 ▸).

After all of the filtering stages, we reduced the number of diffraction patterns from the initial Set_44k_ to 17% with the PCA technique and kept only the high-quality data Set_7k_^PCA^ for further orientation determination. Before orientation determination, we will first compare in detail two data sets: Set_14k_ and Set_7k_^PCA^ by the X-ray cross-correlation analysis (XCCA) approach.

## 5. Angular X-ray cross-correlation analysis   

**Figure 7:**
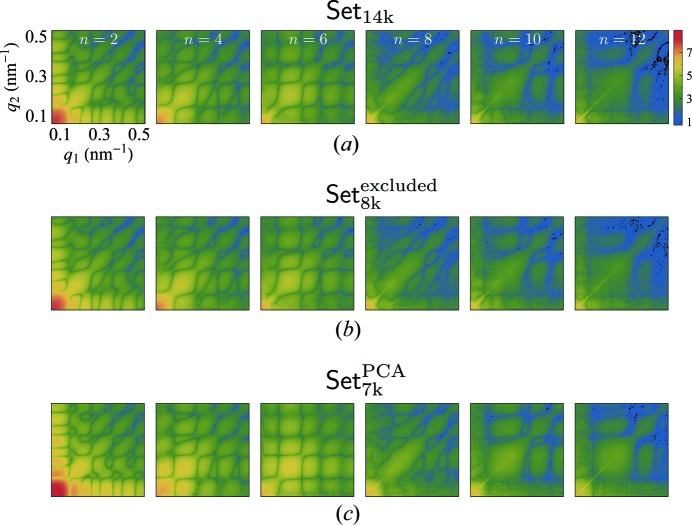
Correlation maps of the amplitudes 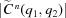 for even order difference spectra Fourier components. (*a*) Set_14k_ and (*b*) Set_8k_^excluded^ have very similar features which suggests that Set_8k_^excluded^ with presumably non-single hits dominates the properties of Set_14k_. (*c*) Set_7k_^PCA^ consists of the PCA single-hit selection and shows more pronounced features.

A two-point angular XCCA  (Altarelli *et al.*, 2010 ▸; Kurta *et al.*, 2013 ▸) was applied to compare our data selection Set_7k_^PCA^ with the Set_14k_^PCA^. The cross-correlation function (CCF) defined here is similar to  Kurta *et al.* (2017 ▸),
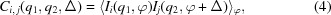
where 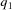 and 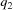 are the momentum-transfer magnitudes at two points with the respective intensity values *I_i_* and *I_j_* of the *i*th and *j*th diffraction pattern, φ and Δ are the angular coordinates and 〈…〉_φ_ denotes angular average. The analysis comprises the Fourier components (FCs) of correlation maps calculated by the ensemble-averaged difference spectra defined as (Kurta *et al.*, 2017 ▸) 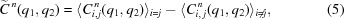
where *C^n^_i,j_*(*q*_1_, *q*_2_) are the FCs of the CCFs of order *n* over the angle Δ and 〈…〉_*i,j*_ denotes the average over diffraction patterns *i* and *j*. The Fourier components here are related to the CCFs in equation (4) by 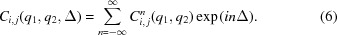
Only the FCs of even orders (*n* = 2; 4; 6; 8; 10; 12) were found to significantly contribute to the difference spectra FCs. In Fig. 7 ▸, we show two-dimensional maps of the amplitudes 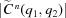 for each FC of order *n* for the data Set_14k_, the excluded data Set_8k_^excluded^ and the PCA selected data Set_7k_^PCA^ (see Fig. 6 ▸).

The correlation-map results of both Set_14k_ and Set_8k_^excluded^ look very similar. This suggests that the data of Set_14k_ is dominated by the contribution of data Set_8k_^excluded^ excluded by the PCA technique.

Although correlation maps are convenient for visual comparison of data sets, the Fourier quadrant correlation (FQC) introduced by Kurta *et al.* (2017 ▸) (see Supporting Information), shows the quantitative similarity of data sets between 0 (no similarity) and 1 (identical) as a function of *q* value.

**Figure 8:**
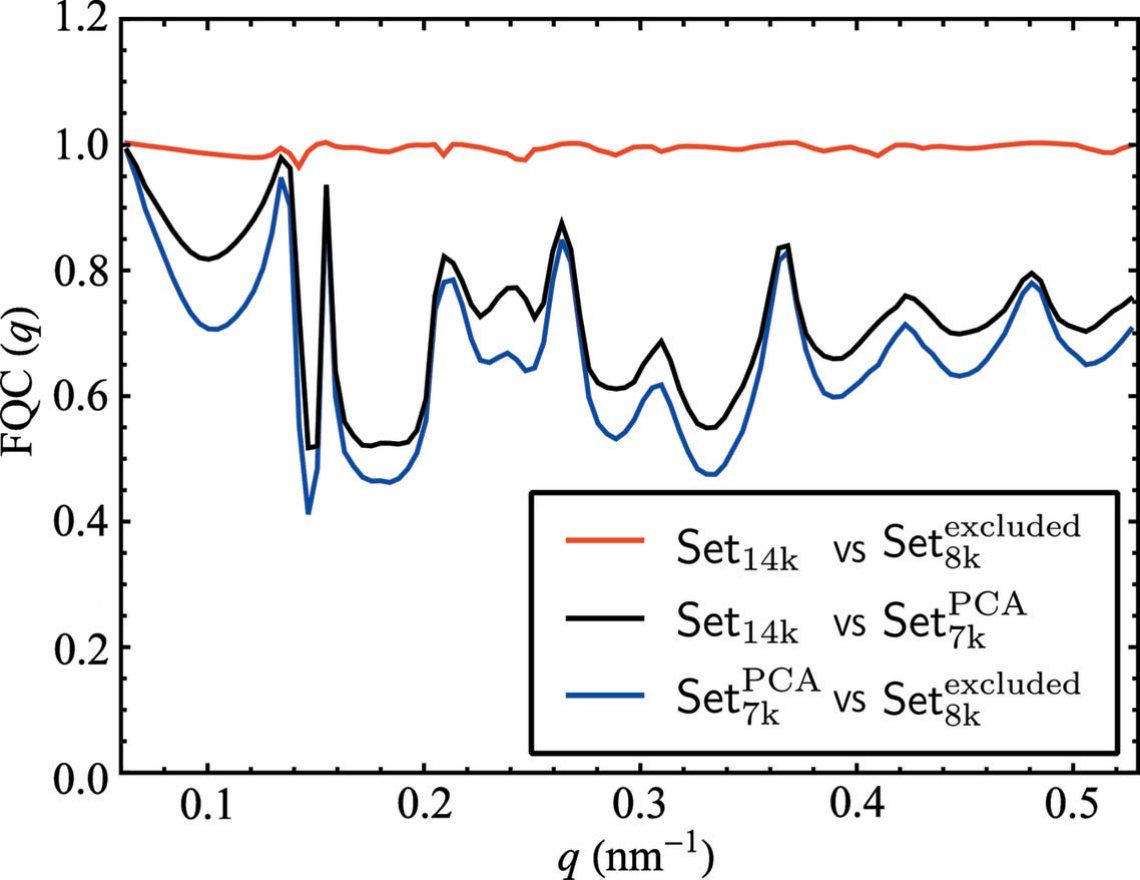
Comparative analysis of different pairs of the selected data sets by the Fourier quadrant correlation (FQC) approach.

We show the FQC for the pairwise comparison between data Set_14k_, Set_8k_^excluded^ and Set_7k_^PCA^ in Fig. 8 ▸. The blue line is the comparison between the PCA selection and the excluded data. We find values much lower than unity at low resolution and conclude on major difference between these two data sets. By comparing the PCA selection and data Set_14k_ (black line), we find the same tendencies with only slightly improved FQC values. This suggests that the data Set_14k_ is heavily influenced by the excluded data and we confirm that by showing the FQC of Set_14k_ and Set_8k_^excluded^, which shows high correlation close to one for all *q* values (red line).

The XCCA and FQC analysis retrieved a quantitative difference between two data sets. This pointed to a strong influence of the excluded data on the whole Set_14k_.

## 6. Angular-orientation determination   

**Figure 9:**
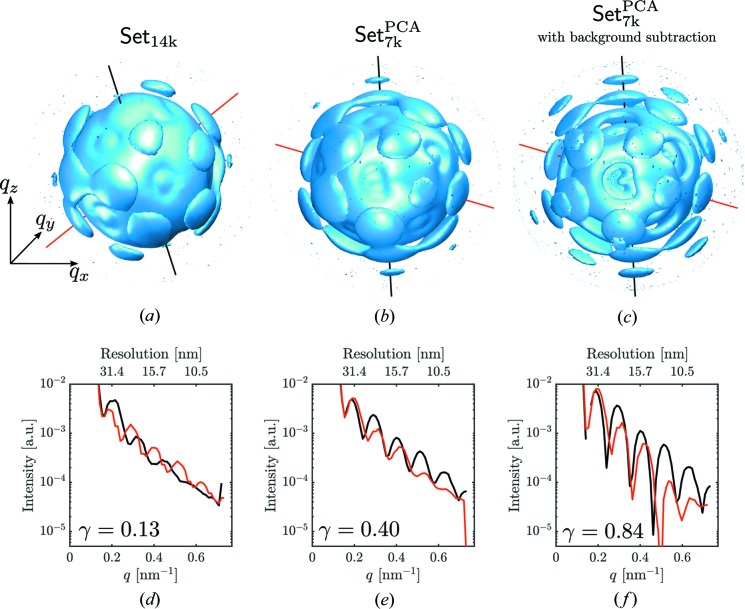
(*a*)–(*c*) Three-dimensional intensity distribution from data Set_14k_ (*a*), data Set_7k_^PCA^ (*b*), and data Set_7k_^PCA^ (*c*) with background subtraction. All three intensity distributions are shown in a logarithmic scale in different orientations. (*d*)–(*f*) Line profiles along the red and black lines shown in (*a*)–(*c*) for three data sets. Note that the data sets in (*a*) and (*b*) are not aligned because of the non-deterministic iterative orientation determination.

For the orientation determination we used the well documented expand–maximize–compress (EMC) algorithm  (Loh & Elser, 2009 ▸) implemented in the software *Dragonfly* (Ayyer *et al.*, 2016 ▸) (see Supporting Information for *Dragonfly* parameters used for orientation determination). For comparison, we retrieved orientations of the selected diffraction patterns for the two data sets: Set_14k_ and Set_7k_^PCA^. The three-dimensional intensity distribution for these two data sets is presented in Figs. 9 ▸(*a*) and 9(*b*). In addition, for the three-dimensional intensity distribution of data Set_7k_^PCA^, the background, in the form of a linear combination of two Gaussian functions, was subtracted [see Fig. 9 ▸(*c*) and the Supporting Information for details].

From the three-dimensional intensity distribution for each data set we conclude that there is strong improvement in the fringe contrast going from the selection Set_14k_ to Set_7k_^PCA^ and further improvement with background subtraction. In Figs. 9 ▸(*d*)–9(*f*) we show line profiles taken in different directions in reciprocal space. To quantify an improvement in contrast from these line profiles we calculate contrast values as 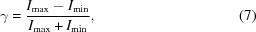
where 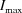 and 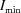 are maximum and minimum values along the line profiles. To characterize each data set with a single number we averaged the contrast values over all neighboring maxima and minima along the line profiles. The diffraction fringe contrast analysis revealed higher contrast of the value 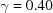 for our data selection Set_7k_^PCA^ over the previously reported data selection Set_14k_ with the value of 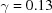. As a consequence of the background subtraction procedure applied to the three-dimensional intensity distribution we obtained a further improved contrast of 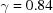.

Line plots of Figs. 9 ▸(*d*)–9(*f*) also show shifted peak positions which indicate a non-symmetric particle shape. Importantly, this would not be possible to observe if symmetry constraints were applied at the angular orientation determination step.

## 7. Electron-density reconstruction   

### 7.1. Virus particle reconstruction   

It is well known that in the frame of kinematical approximation the scattered intensity represents the squared amplitude of the Fourier transform of the three-dimensional electron density 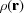 of the particle (Als-Nielsen & McMorrow, 2011 ▸),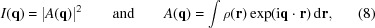 where 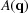 is the scattered amplitude. To determine the three-dimensional electron-density, iterative phase-retrieval tech-niques (Fienup, 1982 ▸; Marchesini, 2007 ▸) may be applied to the three-dimensional intensity distribution of a virus particle determined above. By applying phase retrieval, a three-dimensional electron-density distribution averaged over 100 reconstructions was obtained. We used a combination of algorithms including continuous hybrid input–output (cHIO) (Fienup, 2013 ▸), solvent flipping (SF) (Marchesini, 2007 ▸) and error reduction (ER) (Fienup, 1982 ▸) in combination with shrink-wrap (SW) (Marchesini *et al.*, 2003 ▸). This gave the most stable reconstructions after 1680 iterations from random initial starts (see Supporting Information for details). The averaging ensures the statistical significance from random starts of the reconstruction algorithm.

**Figure 10:**
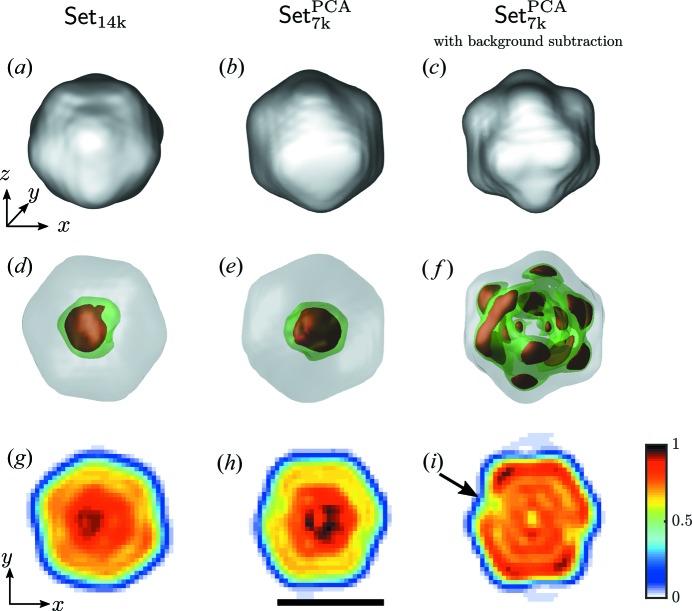
PR772 virus electron-density reconstruction obtained from different data selections. (*a*)–(*c*) Isosurface at 10% of the maximum electron density. (*d*)–(*f*) Isosurface at 10%, 82% and 89% of the maximum electron density. (*g*)–(*i*) Slices through the particle center in the *x*–*y* plane. Low-density site in (*i*) is marked by a black arrow. Black scale bar denotes 50 nm.

Isosurface plots (10% of maximum electron density) of the three-dimensional PR772 virus shape are shown in the first row of Fig. 10 ▸. The second row gives the internal structure at 10%, 82% and 89% isosurface level and the third row comprises slices through the center of the particle reconstructions on the same color scale.

The reconstructed electron density from Set_14k_ contains a small high-density peak in the center of the particle [Figs. 10 ▸(*d*) and 10(*g*)]. The reconstruction from Set_7k_^PCA^ [Figs. 10 ▸(*e*) and 10(*h*)] without background subtraction exhibits a broader central density region. Importantly, the reconstruction with background subtraction from data Set_7k_^PCA^ [see Figs. 10 ▸(*f*) and 10(*i*)] shows the expected density distribution that exhibits the concentric structure with an outer protein capsid and internal lipid membrane surrounding the viral DNA that is characteristic of the Tectiviridae family (Miyazaki *et al.*, 2010 ▸).

From the results of reconstruction we also see that the particle shape deviates from initially expected icosahedral symmetry. The asymmetry is most evident from the background subtracted reconstruction from our selection set [see Fig. 10 ▸(*i*)] and shows a low-density region indicated by a black arrow.

The improvement of the PCA-filtered data with respect to Set_14k_ is seen from the reconstructions. At the same time, background subtraction plays an important role in the final reconstruction result.

### 7.2. Virus size and shape analysis   

The particle electron-density reconstruction was particularly helpful for estimating the virus size and shape. We extracted multiple values for the virus size corresponding to different distances between opposite points of the virus capsid. It is useful to distinguish between the distances from facet to facet and from vertex to vertex. From this we can quantify the shape distortion in various directions.

**Figure 11:**
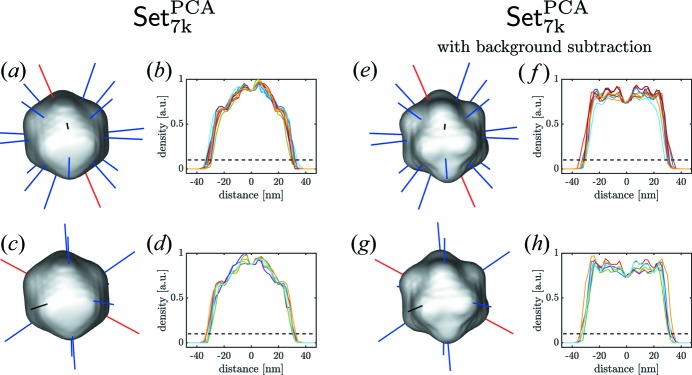
Line scans from facet to facet (top row) and from vertex to vertex (bottom row) in different directions for the selected data set (Set_7k_^PCA^) without (*a*)–(*d*) and with (*e*)–(*h*) background subtraction. The horizontal dotted lines in (*b*), (*d*), (*f*) and (*h*) indicate 10% of the maximum density used for size analysis.

In Fig. 11 ▸ the virus dimensions are analyzed in different directions for both cases of data processing, with and without background subtraction. Each size value corresponds to 10% of the maximum electron-density value as shown by the isosurface. The maximum size of the virus may be estimated as the one measured from vertex to vertex. Our results give for this distance values of 66.2 and 68.5 nm for reconstructions without and with background subtraction, respectively (see Table 2 ▸). This size corresponds very well to an average size of the virus estimated by other means (Reddy *et al.*, 2017 ▸). At the same time for both data sets we observe a pronounced shape distortion of the virus that is about 4.8% and 4.2% of the average virus size for the data sets without and with background subtraction, respectively. This is also consistent with observations made previously (Kurta *et al.*, 2017 ▸), where similar distortion of the virus shape was identified by the XCCA approach.

### 7.3. Resolution estimate   

**Figure 12:**
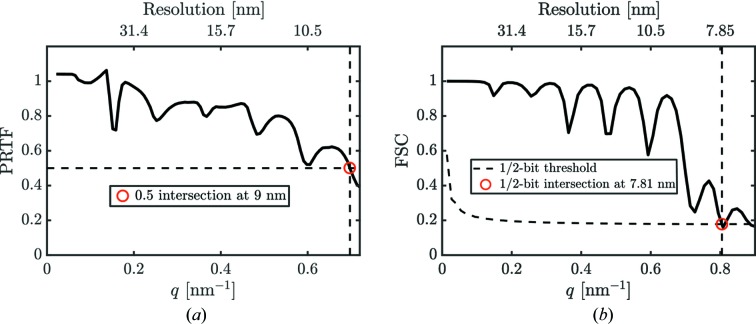
(*a*) Phase-retrieval transfer function (PRTF) and (*b*) Fourier-shell correlation (FSC) of data Set_7k_^PCA^ with background subtraction. Red dots correspond to the determined resolution.

The three-dimensional voxel size of the reconstruction determined by the detector size and experiment geometry was 4.2 nm^3^. Finally, the reconstructed particle volume contained about 17 576 resolution elements (voxels). The first estimate of the resolution in our reconstruction was obtained by the phase-retrieval transfer function (PRTF) (Chapman *et al.*, 2006 ▸) that provided a value of 9 nm at the 0.5 threshold [see Fig. 12 ▸(*a*)]. The second estimate of the resolution was obtained by the Fourier-shell correlation (FSC) analysis (van Heel & Schatz, 2005 ▸). For the FSC analysis the data were split into two parts, each half was oriented independently with EMC and independently reconstructed. The commonly used resolution criterion (van Heel & Schatz, 2005 ▸) of the 1/2-bit threshold line (equal to a signal-to-noise ratio of one in Fourier space of the reconstructed object) intersects the FSC at a resolution value of 7.81 nm. By that analysis we conclude that the resolution of our three-dimensional virus reconstruction presented in Fig. 10 ▸ is in the range 7.8–9 nm and was primarily limited by the detector size.

## 8. Conclusions   

We have presented a workflow from measured single-particle imaging XFEL data to a three-dimensional reconstruction. The workflow consisted of several steps, including single hit diffraction data classification; refined filtering of the classified data; reconstruction of three-dimensional intensity distribution by orientation determination and reconstruction of the particle electron density by phase-retrieval methods (Fig. 1 ▸).

Our research was performed on data taken at the AMO beamline at LCLS as part of the SPI initiative (Reddy *et al.*, 2017 ▸). The analysis was based on initial data selection Set_44k_ of diffraction patterns which were free of obvious faulty data such as empty images. First, using threshold of the diffraction patterns according to their integrated scattered intensity we separated weak single-particle hits from more useful strong hits. Furthermore, diffraction images above a photon count threshold of 2 × 10^5^ ph were classified by the PCA technique. At that step we identified diffraction images as single hits as a result of their clustering into a dense region on the PCA plane.

At the next step, additional refinement filtering was implemented based on comparative analysis of measured diffraction data with the spherical form-factor model. As a result, the final PCA selection after refinement filtering was a fraction of 17% of the initial data and consisted of the Set_7k_^PCA^ of diffraction images.

Furthermore, a newly developed correlation approach based on XCCA was used to compare our data selection with the excluded data of the bigger selection previously reported (Reddy *et al.*, 2017 ▸). Our analysis showed that the selected data were indeed distinct from the excluded data.

The three-dimensional intensity distribution in reciprocal space was determined by the EMC algorithm. In addition, we performed background subtraction from the PCA-selected data set, which substantially improved the contrast of the three-dimensional intensity distribution. At this step we observed a non-symmetric virus shape that would not be possible to identify if symmetry constraints were applied.

At the final step of our workflow we used three-dimensional phase retrieval for the real-space particle reconstruction to reveal the electron density of the measured virus particle. The subsequent size analysis showed a non-symmetric particle shape with an average size of 68.5 nm, and size variation from an ideal icosahedral shape on the order of 4.2%. The resolution was estimated to be better than 10 nm based on PRTF and FSC analyses. With the presented analyses of the reconstructions, we showed that the current limitation on resolution was primarily imposed by the detector size.

Overall, our results demonstrate a feasible approach for analysis of large SPI data sets collected at XFELs. A major observation from our analysis is that PR772 particles do not exhibit true icosahedral symmetry, which is in agreement with analyses of the data set using other approaches  (Kurta *et al.*, 2017 ▸; Hosseinizadeh *et al.*, 2017 ▸). Our analysis shows that particles exhibit a low-density region beneath one of the facets [see Fig. 10 ▸(*i*)]. The biological significance of this remains to be shown, but it is worth noting that the internal membrane of the closely related PRD1 virus undergoes remodeling in response to environmental conditions (like osmolarity) that result in changes to the membrane–capsid interactions (Peralta *et al.*, 2013 ▸). The changes are thought to occur during interactions with the host cell receptor during infection, resulting in destabilization of the icosahedral intermembrane vesicle.

Going forward with XFEL SPI studies, we would like to note that a further increase of the scattering angle in future SPI experiments may be achieved with higher XFEL fluence [with certain limitations imposed by radiation damage  (Lorenz *et al.*, 2012 ▸; Gorobtsov *et al.*, 2015 ▸)]. Importantly, a sufficient number of single hits should be collected in these experiments to produce a useful signal at larger diffraction angles. A substantial enhancement of the hit rate is expected at higher luminosity XFEL facilities such as European XFEL and LCLS II, as well as due to improved sample-injection techniques. We also expect that our PCA technique based on single-hit classification workflow is an important step forward for future data analysis prior to orientation determination and phase retrieval.

## Supplementary Material

Supplementary material containing information on: principal component technique and feature vector compression, angular X-ray cross-correlation analysis, orientation determination and 3D intensity distribution analysis, reconstructions by phase retrieval. DOI: 10.1107/S205225251801120X/it5017sup1.pdf

Supporting information file. DOI: 10.1107/S205225251801120X/it5017sup2.mp4

Supporting information file. DOI: 10.1107/S205225251801120X/it5017sup3.mp4

Supporting information file. DOI: 10.1107/S205225251801120X/it5017sup4.mp4

Supporting information file. DOI: 10.1107/S205225251801120X/it5017sup5.mp4

Supporting information file. DOI: 10.1107/S205225251801120X/it5017sup6.mp4

Supporting information file. DOI: 10.1107/S205225251801120X/it5017sup7.mp4

Supporting information file. DOI: 10.1107/S205225251801120X/it5017sup8.mp4

Supporting information file. DOI: 10.1107/S205225251801120X/it5017sup9.mp4

Supporting information file. DOI: 10.1107/S205225251801120X/it5017sup10.mp4

Supporting information file. DOI: 10.1107/S205225251801120X/it5017sup11.mp4

Supporting information file. DOI: 10.1107/S205225251801120X/it5017sup12.mp4

Supporting information file. DOI: 10.1107/S205225251801120X/it5017sup13.mp4

Supporting information file. DOI: 10.1107/S205225251801120X/it5017sup14.mp4

Supporting information file. DOI: 10.1107/S205225251801120X/it5017sup15.mp4

Supporting information file. DOI: 10.1107/S205225251801120X/it5017sup16.mp4

## Figures and Tables

**Table 1:** Data sets selected for different photon thresholds with the subsequent PCA selection. The percentages of the selected data to Set_44k_ are given in parentheses

Photon threshold	No. of hits	Selected hits	Data set name
Initial set	44 038	–	Set_44k_
> 5 × 10^4^ ph	38 700 (87.9%)	17 337 (39.4%)	Set_17k_^PCA^
> 1 × 10^5^ ph	34 168 (77.6%)	15 229 (34.6%)	Set_15k_^PCA^
> 2 × 10^5^ ph	21 338 (48.5%)	10 082 (22.9%)	Set_10k_^PCA^
			
Size filter	10 082 (22.9%)	8 459 (19.2%)	Set_8k_^PCA^
Fit filter	8 459 (19.2%)	7 303 (16.6%)	Set_7k_^PCA^

**Table 2:** Particle size determined from facet to facet and vertex to vertex distances shown in Fig. 11 ▸. The background subtracted data set is indicated by […]*

		*D*_mean_ (nm)	*D*_max_ (nm) [(*D*_max_/*D*_mean_) − 1]	*D*_min_ (nm) [(*D*_min_/*D*_mean_) − 1]
Set_7k_^PCA^	Facets (b)	62.7	67.5 [7.5%]	58.3 [−7.0%]
	Vertices (d)	63.2	66.2 [4.8%]	60.8 [−3.9%]
				
[Set_7k_^PCA^]*	Facets (f)	63.0	69.0 [9.5%]	60.5 [−4.0%]
	Vertices (h)	65.7	68.5 [4.2%)	62.5 [−5.0%]
