# Supplementary material for: Single-particle imaging without symmetry constraints at an X-ray free-electron laser
Source: IUCrJ. 2018 Sep 18;5(Pt 6):727–36. doi: 10.1107/S205225251801120X (PMC6211532; doi:10.1107/S205225251801120X)
Supplement: Supplementary file 1 [file m-05-00727-sup1.pdf]

IUCrJ

Volume 5 (2018)

Supporting information for article:

Single-particle imaging without symmetry constraints at an X-ray free-electron laser

Max Rose, Sergey Bobkov, Kartik Ayyer, Ruslan P. Kurta, Dmitry Dzhigaev, Young Yong Kim, Andrew J. Morgan, Chun Hong Yoon, Daniel Westphal, Johan Bielecki, Jonas A. Sellberg, Garth Williams, Filipe R.N.C. Maia, Olexander M. Yefanov, Vyacheslav Ilyin, Adrian P. Mancuso, Henry N. Chapman, Brenda G. Hogue, Andrew Aquila, Anton Barty and Ivan A. Vartanyants

Supplemental material: Single particle imaging without symmetry constraints at an X-ray free-electron laser

Max Rose,[a] Sergey Bobkov,[b] Kartik Ayyer,[c] Ruslan P. Kurta,[d] Dmitry Dzhigaev,[a] Young Yong Kim,[a] Andrew J. Morgan,[c] Chun Hong Yoon,[e] Daniel Westphal,[f] Johan Bielecki,[f,d] Jonas A. Sellberg,[g,f] Garth Williams,[h] Filipe R.N.C. Maia,[f,i] Olexander M. Yefanov,[c] Vyacheslav Ilyin,[b,x] Adrian P. Mancuso,[d] Henry N. Chapman,[c] Brenda G. Hogue,[j,k,l] Andrew Aquila,[e*] Anton Barty[c*] and Ivan A. Vartanyants[a,m*]

[a]Deutsches Elektronen-Synchrotron DESY, Notkestraße 85, 22607 Hamburg, Germany, [b]National Research Centre 'Kurchatov Institute', Akademika Kurchatova pl. 1, 123182 Moscow, Russia, [c]Center for Free Electron Laser Science (CFEL), Notkestraße 85, 22607 Hamburg, Germany, [d]European XFEL GmbH, Holzkoppel 4, 22869 Schenefeld, Germany, [e]Linac Coherent Light Source, SLAC National Accelerator Laboratory, 2575 Sand Hill Road, Menlo Park, California 94025, USA, [f]Laboratory of Molecular Biophysics, Department of Cell and Molecular Biology, Uppsala University, Sweden, [g]Biomedical and X-Ray Physics, Department of Applied Physics, AlbaNova University Center, KTH Royal Institute of Technology, Stockholm SE-106 91, Sweden, [h]Brookhaven National Laboratory, 98 Rochester St, Shirley, NY 11967, USA, [i]NERSC, Lawrence Berkeley National Laboratory, Berkeley, California, USA, [j]Biodesign Center for Immunotherapy, Vaccines, and Virotherapy, Biodesign Institute at Arizona State University, Tempe 85287, USA, [k]Biodesign Center for Applied Structural Discovery, Biodesign Institute at Arizona State University, Tempe

85287, USA, [l]*Arizona State University, School of Life Sciences (SOLS), Tempe, Arizona 85287, USA, and* [m]*National Research Nuclear University MEPhI (Moscow Engineering Physics Institute), Kashirskoe shosse 31, 115409 Moscow Russia.*

E-mail: aquila@slac.stanford.edu, anton.barty@cfel.de, ivan.vartaniants@desy.de

1. Principal component technique and feature vector compression

Principal component analysis (PCA) is a well-known approach to reduce the number of data dimensions for efficient analysis of large data sets. However, a direct application of the PCA did not allow to select single hits originating from the diffraction of single particles.

With our PCA technique, we first compress diffraction images into feature vectors (FV), which are based on X-ray cross-correlation analysis and mainly depend on structural features of the particle. The diffraction images depend on intensity fluctuations due to x-ray beam instabilities and the particle position inside the FEL beam. The FV compression mitigates these adverse effects. Each FV shares a one-to-one correspondence with the diffraction image. The FVs are then projected by the PCA onto a plane.

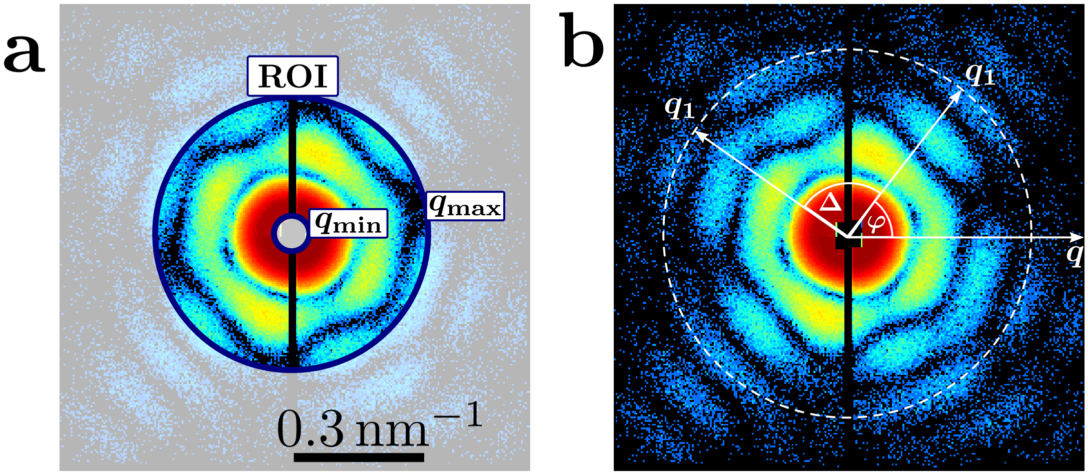

Fig. 1. Polar coordinate system in the diffraction plane. a) Conventions for the correlation analysis and b) Region of interest (ROI) of the feature vector definition.

Here we describe the general procedure for FV construction. Information in a diffraction pattern is distributed non-uniformly. We select a region of interest (ROI) in the form of an annulus $q_{\min} \leq q \leq q_{\max}$ as shown in Figure 1 a. The value of $q_{\min} = 0.05\,\mathrm{nm}^{-1}$ was set to avoid the rectangular beam stop area (see Figure 1 b). The value of $q_{\max} = 0.26\,\mathrm{nm}^{-1}$ was set to one half of the distance from the center to the edge of detector. At $q > q_{\max}$ the measured intensity was substantially lower and the impact on the FVs from this area were dominated by noise. Note that the same ROI was used for all diffraction images of data $\mathsf{Set}_{44\mathrm{k}}$.

For each image we compute correlation coefficients

$$C(q,\Delta) = \langle I(q,\varphi) I(q,\varphi+\Delta)\rangle_{\varphi}\ , \tag{1}$$

as the angular average denoted by $\langle \ldots \rangle_{\varphi}$ within the ROI. Here q is the radial coordinate on the polar grid of a diffraction image, φ and Δ are the angular coordinates as shown in Figure 1 b. To reduce the parameter space of the correlation coefficients also the average over the radial coordinate $\langle \ldots \rangle_q$ is calculated by

$$C(\Delta) = \langle C(q,\Delta)\rangle_q\ . \tag{2}$$

With the normalization of equation (2) by the averaged ROI intensity

$$\bar{C}(\Delta) = \frac{C(\Delta)}{\langle I(q,\varphi)^2 \rangle_{\varphi,q}} = \frac{C(\Delta)}{C(0)} \ . \tag{3}$$

The normalization by intensity reduces the effect of intensity fluctuations.

After that, the function $\bar{C}(\Delta)$ defined in Eq. (3) was expanded into Fourier cosine series,

$$\bar{C}(\Delta) = 2 \sum_{n=1}^{\infty} \bar{C}^n \cos(n\Delta) \ , \tag{4}$$

$$\bar{C}^n = \frac{1}{\pi} \int_0^{\pi} \bar{C}(\Delta) \cos(n\Delta) \, \mathrm{d}\Delta \ , \tag{5}$$

where $\bar{C}^n$ are the Fourier components (FC) of $\bar{C}(\Delta)$. The FCs $\bar{C}^n$ constitute a compact set of parameters that carry information on angular features of the diffraction patterns. Typically, a limited number m of nonzero FCs contributes to the spectrum $\bar{C}^n$, depending on the particle structure and experimental conditions (Kurta *et al.*, 2016; Kurta *et al.*, 2013). The number m can be chosen much smaller than the total number of pixels on the detector with $M = 66\,820$. From the data we concluded that only the first $m \approx 20$ FCs substantially differ from zero. We set $m = 50$ to be sure that to include all valuable information on angular features. The feature vector $\boldsymbol{FV}$ of a diffraction pattern was then defined as

$$\boldsymbol{FV} = (\bar{C}^1, \ldots, \bar{C}^m). \tag{6}$$

Each FV is one-dimensional with a length of 50 elements. With this definition FVs are determined primarily by the particle structure.

1.1. Selected data

In Table 1 we give references to the original data used for the PCA technique. For visual inspection of the data we show the 100 brightest and weakest diffraction patterns as example in animations.

Table 1. *File references for measured data and data selection obtained by the PCA technique (Yoon, 2017; Rose, 2018).*

Set_{44k}		Set_{14k}	
data file name	file size	data file name	file size
amo86615_182_PR772.h5	390 MB	singleHitList.h5	777 KB
amo86615_183_PR772.h5	507 MB	references of 14772 hits in Set_{44k}	
amo86615_184_PR772.h5	343 MB		
amo86615_185_PR772.h5	336 MB		
amo86615_186_PR772.h5	8.7 GB		
amo86615_188_PR772.h5	7.6 GB		
amo86615_190_PR772.h5	5.9 GB		
amo86615_191_PR772.h5	5.5 GB		
amo86615_192_PR772.h5	4.4 GB		
amo86615_193_PR772.h5	7.3 GB		
amo86615_194_PR772.h5	4.8 GB		
amo86615_196_PR772.h5	5.6 GB		
amo86615_197_PR772.h5	12 GB		
		animation file name	file size
		brightestHits_amo86615_PR772_14k.mp4	1.19 MB
		weakestHits_amo86615_PR772_14k.mp4	1.19 MB
$\mathsf{Set}_{7k}^{\mathrm{PCA}}$		$\mathsf{Set}_{8k}^{\mathrm{excluded}}$	
data file name	file size	data file name	file size
amo86615_PR772_7k_PCA_7303.h5	1.01 GB	amo86615_PR772_8k_excluded_8095.h5	1.1 GB
animation file name	file size	animation file name	file size
brightestHits_amo86615_PR772_7k.mp4	1.19 MB	brightestHits_amo86615_PR772_8k.mp4	1.19 MB
weakestHits_amo86615_PR772_7k.mp4	1.19 MB	weakestHits_amo86615_PR772_8k.mp4	1.19 MB
		image file name	file size
		amo86615_PR772_8k_excluded_8095_examples.pdf	225 kB
$\mathsf{Set}_{6.6k}^{\mathrm{in\ common}}$		$\mathsf{Set}_{7k}^{\mathrm{PCA}} \notin \mathsf{Set}_{14k}$	
data file name	file size	data file name	file size
amo86615_PR772_6.6k_PCA_6677.h5	956 MB	amo86615_PR772_7k_NOTin_14k.h5	184 MB
animation file name	file size	animation file name	file size
brightestHits_amo86615_PR772_6.6k.mp4	1.19 MB	brightestHits_amo86615_PR772_7k_NOTin_14k.mp4	1.19 MB
weakestHits_amo86615_PR772_6.6k.mp4	1.19 MB	weakestHits_amo86615_PR772_7k_NOTin_14k.mp4	1.19 MB

2. Angular X-ray cross-correlation analysis

While the correlation maps are convenient for visual comparison of data sets, the Fourier quadrant correlation (FQC) (Kurta *et al.*, 2017)

$$\mathrm{FQC}(q) = \frac{1}{N(q)} \left(\sum_{q_1 \leq q} CC(q_1, q_2) + \sum_{q_2 < q} CC(q, q_2) \right), \tag{7}$$

shows the quantitative similarity of data sets between 0 (no similarity) and 1 (identical) as a function of q-value, where the summations are performed over the edges of quadrants $N(q) = \sum_{q_1 \leq q} 1 + \sum_{q_2 < q} 1$. The FQC definition comprises the cumulative correlation metric

$$CC(q_1, q_2) = \frac{CC_{1,2}(q_1, q_2)}{\sqrt{CC_{1,1}(q_1, q_2) CC_{2,2}(q_1, q_2)}}, \tag{8}$$

that include the pairwise correlation of difference CCF from equation (5) in the main text with

$$CC_{v,w}(q_1, q_2) = \langle \widetilde{C}_v(q_1, q_2, \Delta) \widetilde{C}_w(q_1, q_2, \Delta) \rangle_\Delta, \quad (9)$$

between two datasets $v, w = 1, 2$.

A previously selected data set based on $\pm 3\,\mathrm{nm}$ particle size distribution with 566 diffraction patterns (Kurta *et al.*, 2017) was used for a comparative evaluation with the PCA selected data. We have calculated the difference of correlation features to the $\mathsf{Set}_{7k}^{\mathrm{PCA}}$ and found good similarity as indicated by FQC values everywhere above 0.75 in Figure 2. However both data selections are not exactly likewise and may explain also the differences in the final particle reconstructions results compared to (Kurta *et al.*, 2017).

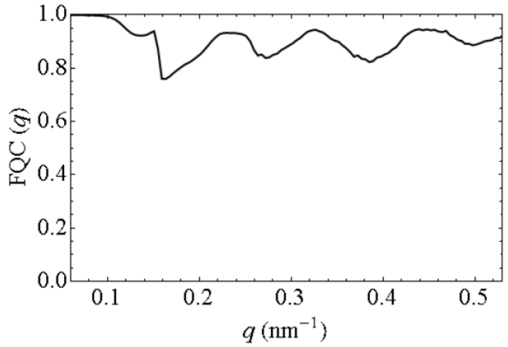

Fig. 2. Similarity analysis by Fourier quadrant correlation (FQC) between PCA selected data $\mathsf{Set}_{7k}^{\mathrm{PCA}}$ and size distribution limited data of 566 diffraction patterns (Kurta *et al.*, 2017).

3. Orientation determination and 3D intensity distribution analysis

The orientation determination has been performed for both data sets Set_{14k} and $\mathsf{Set}_{7k}^{\mathrm{PCA}}$ with the same parameters. The `config.ini` file (see Table 2) contains all important EMC parameters used for the EMC process of Dragonfly (Ayyer *et al.*,

2016). After 100 iterations we stopped the EMC algorithm as the diffraction volume has been shaped to a reasonable solution. Animations of the iterative orientation determination are referenced in Table 2. By inspection of the RMS error change in Figure 3 a we find a plateau beyond iteration 50, suggesting convergence of the orientation determination. Vertical dotted lines indicate the beta value increment by factor $\sqrt{2}$. An increase every 10 iterations helps to overcome known stagnation problems of EMC and gradually sharpens the orientation probability range of each diffraction pattern (Ayyer *et al.*, 2016). Finally we observe an oscillatory RMS change beyond iteration 80 and the diffraction volume leaves the successful orientation solution already found. The steady state between iteration 50 and 80 is seen in the animation (`7k_bgSub_EMC_iterations_3D.mp4 & *2D.mp4` see Table 2). As a result we used the EMC output at iteration 80 for background subtraction and 3D phase retrieval.

In order to make the orientation determination robust against the background that leaks through cleaning apertures in the center of each diffraction pattern a mask (`mask_pnccd_back_260_257.byt` see Table 2) that defines good (0), passive (1) and bad pixels (2) as shown in Figure 3 b. Good pixels are used for orientation, passive pixels are not used for the orientation but will still be merged into the diffraction volume and bad pixels are completely ignored by the EMC algorithm (Ayyer *et al.*, 2016).

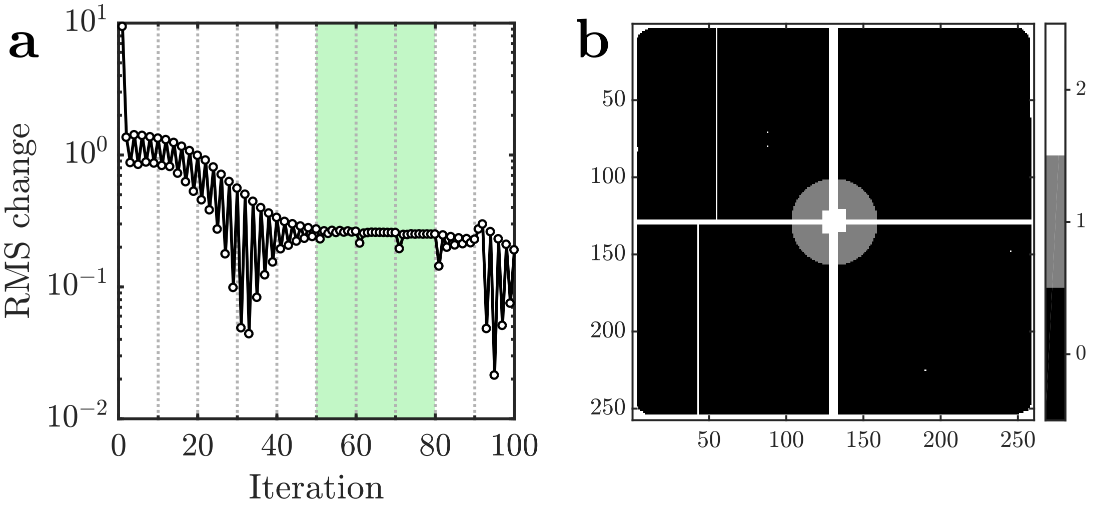

Fig. 3. a) RMS error as a function of EMC iteration number. The result from EMC at iteration 80 was used for phase retrieval. b) Mask of the detector pixels used for orientation determination. Three pixel categories are available: Good (0), passive (1), bad (2).

3.1. Facet intensity modulation

As was shown in the main text of section 7 the diffraction volume is not symmetric. In order to visualize the particle diffraction volume an animation through increasing isosurface values illustrates the complicated deformation in all directions (see animation `7k_bgSub_3D_variable_isosurface.mp4` in Table 2).

In Figure 9 d – f of the main text we showed the specific fringe modulation in two distinct directions to emphasize the shape deformation seen from the diffraction volumes. Here we show in Figure 4 the average fringe contrast taken from intensities corresponding to all facet normals of the particle to support our finding of overall better data quality for the PCA selected data Set_{7k}^{PCA} after orientation determination by EMC.

The average and the range between minimum and maximum modulation is plotted for direct comparison for both data Set_{14k} and Set_{7k}^{PCA}. Clearly the average modulation

of data $\mathsf{Set}_{7\mathrm{k}}^{\mathrm{PCA}}$, indicated by the red line is more pronounced than for the data $\mathsf{Set}_{14\mathrm{k}}$, indicated by the blue line. The same result is found for the ranges of minimum and maximum fringe modulation indicated by the light red and light blue patches in Figure 4. The sub-figures a & b of Figure 4 refer to the cases without and with background subtraction, respectively.

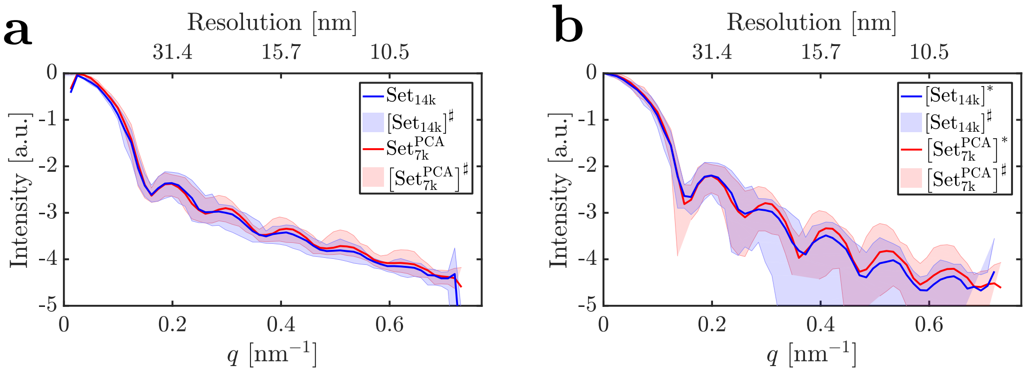

Fig. 4. Modulation of diffraction intensity along particle facet normals. The mean modulation is indicated as lines and the range of values in all directions as colored areas denoted by $[\ldots]^\sharp$. a) Data without and b) with background subtraction denoted by $[\ldots]^*$.

3.2. Background correction

In order to determine the background level we compare the PSD (equation (1) in the main text) obtained from our selection of the measured data shown in Figure 9 b of the main text and the PSD corresponding to a spherical particle (red line in Figure 5). Both PSD curves are normalized to the first side maximum at $q \approx 0.2\,\mathrm{nm}^{-1}$. It is well seen that the contrast between maxima and minima is still rather low for the selected data set $\mathsf{Set}_{7\mathrm{k}}^{\mathrm{PCA}}$ (empty black diamonds). We also observe that the central maximum height of the measurement deviates from the model curve (compare red line with empty black diamonds in Figure 5) due to residual background. Fitting the difference between model and measured data with two Gaussian functions we obtain

an effective background (dashed green line). The background function is defined as follows

$$B(q) = \alpha G_1(q) + \beta G_2(q), \tag{10}$$

where

$$G_{1,2}(q) = \left(2\pi\sigma_{1,2}^2\right)^{-\frac{3}{2}} \exp\left(-\frac{q^2}{2\sigma_{1,2}^2}\right) \tag{11}$$

with fit parameters $\sigma_1 = 23\,\mathrm{nm}^{-1}$, $\sigma_2 = 9\,\mathrm{nm}^{-1}$ and $\alpha = 4.5 \times 10^{-4}$, $\beta = 1.8 \times 10^{-3}$. The two Gaussian functions can be interpreted as G_1 being responsible for the low-q background and G_2 for the high-q background contribution.

The PSD of the background subtracted data (blue line with dots) shows a larger fringe contrast and was similar to the sphere model PSD with respect to the maxima positions. The effect of background subtraction with increased fringe contrast is also presented in the 3D diffraction volume and line scans of Figure 8 c & f in the main text .

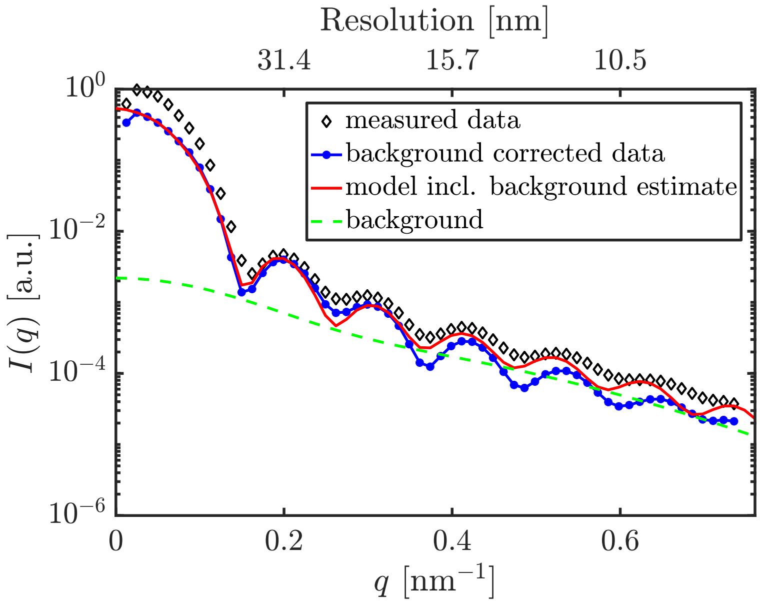

Fig. 5. PSD curves of the measured data, sphere model and background corrected data. The background estimate is shown for comparison.

The background correction applied to the diffraction volume after EMC orientation determination shows good results for the PCA technique selection $\mathsf{Set}_{7\mathrm{k}}^{\mathrm{PCA}}$ but creates an disturbing artifact for $\mathsf{Set}_{14\mathrm{k}}$. In Figure 6 we compare the cases with and without background subtraction for both data sets. The cross sections through the 3D reciprocal intensity are shown. The pixels in the center marked by white color correspond to the missing intensity values behind the beam stop (note that the diffraction volume contains interpolated intensity values and the beam stop region occupies less pixels as compared to the diffraction data, see Figure 3 b. While the data sets without background subtraction Figure 6 a & c look similar are siginifcant difference can be seen in the background subtracted data in Figure 6 b & d. In case of the previously reported data selection $\mathsf{Set}_{14\mathrm{k}}$ an rectangular artifact is visible after background subtraction. It is not exactly clear what causes this artifact because the image in Figure 6 b is the result of orientation determination by EMC. However, we found as a major difference, that $\mathsf{Set}_{14\mathrm{k}}$ contained diffraction data with an imprint of the cleaning apertures used to prevent high-q background scattering from illuminating the detector.

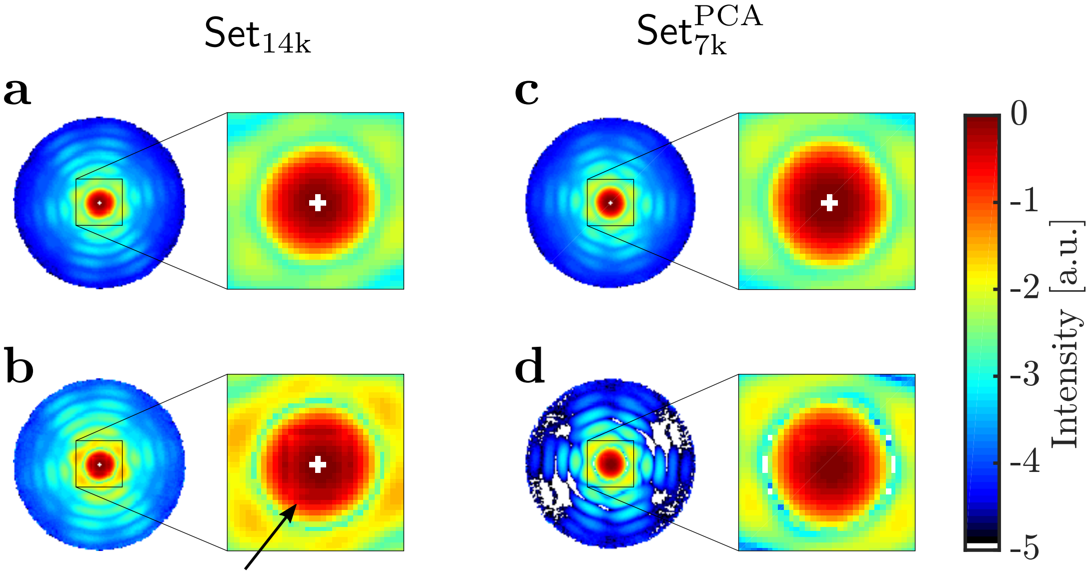

Fig. 6. EMC results of orientation determination with a) & b) and without background subtraction c) & d). Note the missing central values were filled by linear interpolation in the volume. The enlarged region in b) shows the cleaning aperture effect in the central fringe indicated by the black arrow. All slices through the volumes are shown on a logarithmic scale.

In Table 2 we reference the diffraction volumes obtained from EMC with and without background subtraction. In addition we reference also half data sets of $\mathsf{Set}_{7\mathrm{k}}^{\mathrm{PCA}}$ used for FSC analysis. All data files listed in Table 2 are mat-files of version '-v7.3' and can be opened i.e. with *Matlab 2006b* or higher or by using the *h5py* module in python.

Table 2. *File references of diffraction volumes obtained by EMC (Rose, 2018).*

**EMC parameter files**		**EMC iterations**	
data file name	file size	animation file name	file size
config.ini	1 kB	7k_bgSub_EMC_iterations_3D.mp4	3.5 MB
mask_pnccd_back_260_257.byt	66 kB	7k_bgSub_EMC_iterations_2D.mp4	337 kB
		7k_bgSub_EMC_iterations_3D_transparent.mp4	2.2 MB
**Set$_{14k}$**		**Set$_{7k}^{PCA}$**	
data file name	file size	data file name	file size
14k_v7.3.mat	6.4 MB	7k_v7.3.mat	6.7 MB
14k_bgSub_v7.3.mat	6.4 MB	7k_bgSub_v7.3.mat	6.1 MB
animation file name	file size	animation file name	file size
14k_v7.3.mp4	15.4 MB	7k_v7.3.mp4	17.9 MB
14k_bgSub_v7.3.mp4	7.4 MB	7k_bgSub_v7.3.mp4	6.1 MB
**Set$_{7k}^{PCA}$ 1st − half**		**Set$_{7k}^{PCA}$ 2nd − half**	
data file name	file size	data file name	file size
7k_1stHALF_v7.3.mat	6.7 MB	7k_2ndHALF_v7.3.mat	6.7 MB
7k_1stHALF_bgSub_v7.3.mat	6.2 MB	7k_2ndHALF_bgSub_v7.3.mat	5.9 MB
animation file name	file size	animation file name	file size
7k_1stHALF_v7.3.mp4	20.2 MB	7k_2ndHALF_v7.3.mp4	18.3 MB
7k_1stHALF_bgSub_v7.3.mp4	35.6 MB	7k_2ndHALF_bgSub_v7.3.mp4	31.9 MB

4. Reconstructions by phase retrieval

We have used a phase retrieval procedure based on the continuous hybrid input output algorithm (cHIO 30 iterations with feedback value of 0.8), the solvent flipping algorithm (SF, 500 iterations with feedback value of 0.8) and error reduction (ER, 30 iterations) in combination with the shrink-wrap algorithm (every 10-th iteration with threshold value of 0.15 and gaussian filtering of the support with 2 to 0.5 sigma). This combination of algorithms was used three times in succession until the resontruction result was saved. We performed 100 reconstructions and took the average as the final result.

As result many individual reconstructions from random initial starting guesses were averaged using the global phase adjustment (real part maximization) described in (Chapman *et al.*, 2006). This ensures that the complex valued reconstruction results add up coherently for an improved average result. Each individual reconstruction was flipped over three perpendicular axes and aligned respectively to the first reconstruction (flip correction). In addition the individual reconstructions were centered (center

of mass) in space. All individual reconstructions as well as the averaged results are referenced in Table 3. All data is saved in mat-files of version '-v7.3'.

Table 3. *File references for phase retrieval reconstruction results (Rose, 2018).*

Set_{14k}		Set_{7k}^{PCA}	
data file name	file size	data file name	file size
14k_averaged_v7.3.mat	256 kB	7k_averaged_v7.3.mat	230 kB
		7k_individual_v7.3	221 MB
		7k_bgSub_averaged_v7.3.mat	231 kB
		7k_bgSub_individual_v7.3.mat	164 MB
animation file name	file size	animation file name	file size
14k_averaged_OuterShell.mp4	3.6 MB	7k_averaged_OuterShell.mp4	3.7 kB
14k_averaged_slices.mp4	2.1 MB	7k_averaged_slices.mp4	1.9 MB
		7k_bgSub_averaged_OuterShell.mp4	3.7 kB
		7k_bgSub_averaged_slices.mp4	1.9 MB
		7k_bgSub_fixed_isosurface_rotation.mp4	13.3 MB
		7k_bgSub_variable_isosurface_rotation.mp4	25.6 MB
Set_{7k}^{PCA} $\mathbf{1^{st} - half}$		Set_{7k}^{PCA} $\mathbf{2^{nd} - half}$	
data file name	file size	data file name	file size
7k_Half1_bgSub_individual_v7.3.mat	71 MB	7k_Half2_bgSub_individual_v7.3.mat	72 MB
7k_Half1_bgSub_averaged_.mat	247 kB	7k_Half2_bgSub_averaged_v7.3.mat	249 kB
7k_Half1_bgSub_averaged_aligned_v7.3.mat	263 kB	7k_Half2_bgSub_averaged_aligned_v7.3.mat	254 kB
animation file name	file size	animation file name	file size
7k_Half1_bgSub_averaged_OuterShell.mp4	3.8 MB	7k_Half2_bgSub_averaged_OuterShell.mp4	4.0 MB
7k_Half1_bgSub_averaged_slices.mp4	2.6 MB	7k_Half2_bgSub_averaged_slices.mp4	2.6 MB
7k_Half1_bgSub_averaged_aligned_OuterShell.mp4	3.8 MB	7k_Half2_bgSub_averaged_aligned_OuterShell.mp4	4.1 MB
7k_Half1_bgSub_averaged_aligned_slices.mp4	2.1 MB	7k_Half2_bgSub_averaged_aligned_slices.mp4	2.6 MB

To check the reliability of the retrieved average reconstruction results, we plot the histogram of standard deviations of all voxel values obtained from the individual reconstructions of data Set_{7k}^{PCA} with and without background subtraction in Figure 7. The interpretation is that the normalized electron density values inside the virus volume fluctuate much less in case of the background subtraction compared to the case without background subtraction. The central part of the virus having approximately a normalized electron density of 0.7 has a maximum standard deviation between 0.3 and 0.4 when using the background subtracted data.

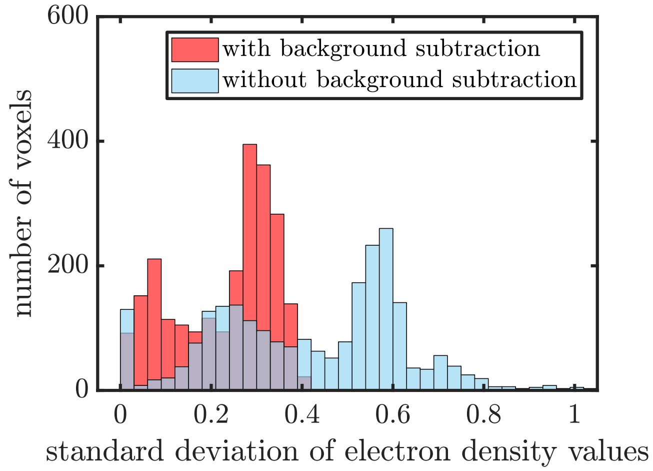

Fig. 7. The histogram of normalized (from 0 to 1) electron density for the individual reconstructions of data Set_{7k}^{PCA} with and without background subtraction.

For the resolution estimation by the FSC a data set is split into two halves. For each half EMC was run independently. Background subtraction was also performed for each half separately. Here we show the averaged reconstructions from the half data sets of Set_{7k}^{PCA} in Figure 8. Both reconstructions are not oriented likewise due to the individual EMC runs. However both average results show similar features surch as the overall shape and the low density part in the virus capsid (indicated in Figure 8 by the black arrows).

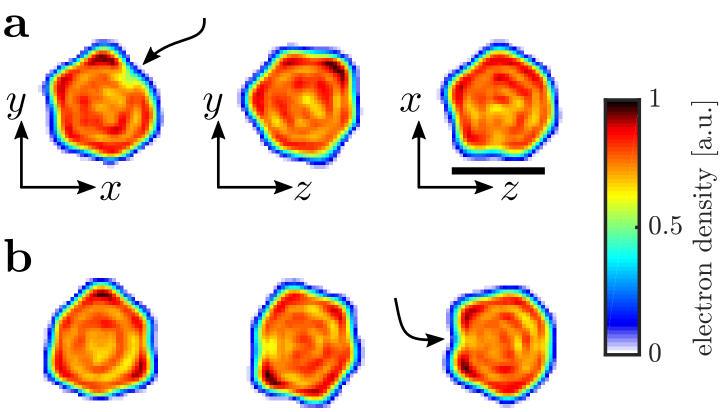

Fig. 8. Particle reconstructions (without alignment) of half data sets from Set_{7k}^{PCA}: a) 1st – half and b) 2nd – half.

In order to compare how well the missing phases were retrieved we show the PRTF for all average results reconstructions based on the Set_{7k}^{PCA} with background subtraction. The half data sets show similar behavior and resolution compared to the full data set.

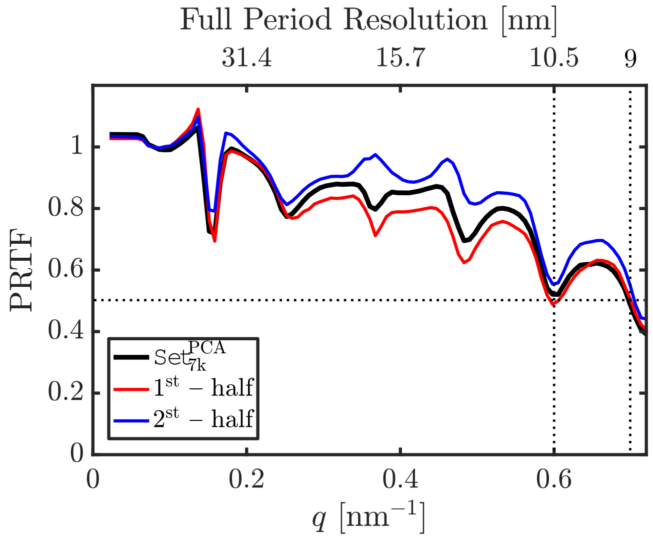

Fig. 9. PRTF for all reconstructions presented in this work. Only the 1st – half cuts the threshold at 10.5 nm. The other half and the full data Set_{7k}^{PCA} intersect at 9 nm.

References

Ayyer, K., Lan, T.-Y., Elser, V. & Loh, N. D. (2016). *Journal of Applied Crystallography*, **49**(4), 1320–1335.

Chapman, H. N., Barty, A., Marchesini, S., Noy, A., Hau-Riege, S. P., Cui, C., Howells, M. R., Rosen, R., He, H., Spence, J. C. H., Weierstall, U., Beetz, T., Jacobsen, C. & Shapiro, D. (2006). *Journal of the Optical Society of America A*, **23**(5), 1179–1200.

Kurta, R., Dronyak, R., Altarelli, M., Weckert, E. & Vartanyants, I. (2013). *New journal of physics*, **15**(1), 013059.

Kurta, R. P., Altarelli, M. & Vartanyants, I. A. (2016). In *Advances in Chemical Physics*, pp. 1–39. John Wiley & Sons, Inc.

Kurta, R. P., Donatelli, J. J., Yoon, C. H., Berntsen, P., Bielecki, J., Daurer, B. J., DeMirci, H., Fromme, P., Hantke, M. F., Maia, F. R., Munke, A., Nettelblad, C., Pande, K., Reddy, H. K., Sellberg, J. A., Sierra, R. G., Svenda, M., van der Schot, G., Vartanyants, I. A., Williams, G. J., Xavier, P. L., Aquila, A., Zwart, P. H. & Mancuso, A. P. (2017). *Physical Review Letters*, **119**(15).

Rose, M. (2018). *Coherent X-ray Imaging Data Bank.* CXIDB ID 88 http://dx.doi.org/10.11577/1471489.

Yoon, C. H. (2017). *Coherent X-ray Imaging Data Bank.* CXIDB ID 58 http://dx.doi.org/10.11577/1349664.
